# The relationship between community public health, behavioral health service accessibility, and mass incarceration

**DOI:** 10.1186/s12913-022-08306-6

**Published:** 2022-07-29

**Authors:** Niloofar Ramezani, Alex J. Breno, Benjamin J. Mackey, Jill Viglione, Alison Evans Cuellar, Jennifer E. Johnson, Faye S. Taxman

**Affiliations:** 1 Department of Statistics, School of Computing, George Mason University, 4400 University Drive, Fairfax, VA MS 4A7 USA; 2 Center for Advancing Correctional Excellence, Schar School of Policy and Government, George Mason University, Fairfax, VA USA; 3 Department of Criminal Justice, University of Central Florida, Orlando, FL USA; 4 Department of Health Administration and Policy, George Mason University, Fairfax, VA USA; 5 Division of Public Health, Michigan State University, Flint, MI USA

**Keywords:** Jail population, Access to care, Community service accessibility, Behavioral health, Mass incarceration, Community public health

## Abstract

**Background:**

The relationship between healthcare service accessibility in the community and incarceration is an important, yet not widely understood, phenomenon. Community behavioral health and the criminal legal systems are treated separately, which creates a competing demand to confront mass incarceration and expand available services. As a result, the relationship between behavioral health services, demographics and community factors, and incarceration rate has not been well addressed. Understanding potential drivers of incarceration, including access to community-based services, is necessary to reduce entry into the legal system and decrease recidivism. This study identifies county-level demographic, socioeconomic, healthcare services availability/accessibility, and criminal legal characteristics that predict per capita jail population across the U.S. More than 10 million individuals pass through U.S. jails each year, increasing the urgency of addressing this challenge.

**Methods:**

The selection of variables for our model proceeded in stages. The study commenced by identifying potential descriptors and then using machine learning techniques to select non-collinear variables to predict county jail population per capita. Beta regression was then applied to nationally available data from all 3,141 U.S. counties to identify factors predicting county jail population size. Data sources include the Vera Institute’s incarceration database, Robert Wood Johnson Foundation’s County Health Rankings and Roadmaps, Uniform Crime Report, and the U.S. Census.

**Results:**

Fewer per capita psychiatrists (z-score = -2.16; *p* = .031), lower percent of drug treatment paid by Medicaid (-3.66; *p* < .001), higher per capita healthcare costs (5.71; *p *< .001), higher number of physically unhealthy days in a month (8.6; *p* < .001), lower high school graduation rate (-4.05; *p* < .001), smaller county size (-2.66, *p* = .008; -2.71, *p* = .007; medium and large versus small counties, respectively), and more police officers per capita (8.74; *p* < .001) were associated with higher per capita jail population. Controlling for other factors, violent crime rate did not predict incarceration rate.

**Conclusions:**

Counties with smaller populations, larger percentages of individuals that did not graduate high school, that have more health-related issues, and provide fewer community treatment services are more likely to have higher jail population per capita. Increasing access to services, including mental health providers, and improving the affordability of drug treatment and healthcare may help reduce incarceration rates.

## Background

As of 2019, 10.3 million people are admitted to U.S. jails per year [66]. Jails are known for housing a high concentration of individuals with mental illness: 45 percent of the jail population reported a history of mental health (MH) issues, with nearly half reporting serious psychological distress in the past 30 days [7]. The percentage of individuals held in jail who meet threshold criteria for serious psychological distress (26%) is over five times greater than the general population of non-incarcerated adults (5%) [7].﻿ The overall capacity for community-based mental health services and size of the jail can be affected by an array of geographic and socio-economic factors (e.g., poverty, income inequality, rurality, etc.) [3, 48]. However, the interplay between the size of jail populations and community-level behavioral health services (i.e., mental health, substance use, etc.) has not been rigorously studied. This study examines how socio-economic, health, behavioral health, and justice factors are related to per capita jail population in U.S. counties.

### Jails in the U.S.

The United States is often cited as an extreme case when it comes to incarceration, with an incarceration rate that is more than three times higher than comparable nations such as Canada, Australia, or England [27, 62]. Carceral practices in the U.S. are closely tied to social and economic marginalization.

Many have argued that incarceration exists not as a response to crime, but as an institution tasked with “governing social marginality” [5, 42], including supplementing or replacing social welfare institutions with penal ones [5, 32, 61]. Others have argued that incarceration in the U.S. largely supplanted hospitalization as a policy response to mental illness in the wake of widespread dinstitutionalization of mental health services beginning in the 1960s [61]. As of 2014, there were around 10 times as many individuals with serious mental illness in prisons and jails than in state psychiatric hospitals [50, 51]. Individuals in U.S. jails and prisons experience serious psychological distress at a rate three to five times greater than the general population [7], yet access to substance use and mental health care in institutional settings is very low [45]. The most recent data available suggests that of those with a history of mental illness, 63% in prisons and 45% in jails received some treatment since being admitted, with less than a quarter of people incarcerated in jail receiving any type of counseling or therapy services [7]. Of those with serious psychological distress, only 54% of those in prison and 35% of those in jail received any type of treatment since admission [7].

The incarceration of individuals with mental illness is common, and generally due to the inability to pay for bail, a lack of stable housing, and other sociostructural factors that affect the likelihood that an individual will be incarcerated [41, 46]. As noted by Winkler and colleagues [63], these structural factors are the result of the escalation of the war on drugs and changes in housing and employment markets that made it difficult to pursue welfare responses to mental health needs, to name a few. Jails differ from prisons in the U.S. in that they house individuals for pretrial detention (more than 10 million per year; median stay of less than a week) and individuals sentenced to less than 1 year of incarceration (about 1 million per year), and they tend to be administered at the county or municipal level rather than at the state or federal leve﻿l [10]. Jails are the primary setting where individuals experience incarceration [52], and jails are particularly chaotic environments that tend to rapidly process individuals while lacking stable services and treatment availability as well as funding for behavioral health services [31, 46]. Community capacity to treat behavioral health disorders is extremely low [46].

Besides limited access to care, the experience of incarceration may exacerbate behavioral health conditions [53, 65]. Jail incarceration may detrimentally impact the physical and behavioral health of a community [24, 25, 35]. Recent estimates indicate that as increases occur in jail incarceration rate, increases also occur in county-wide mortality from substance use and suicide, as well as a range of diseases and injuries [24]. Justice-involved individuals have much higher rates of suicide risk, major depression, substance use disorder, and bipolar disorders than the general population [30, 34, 38, 43, 65]. This suggests that high rates of jail incarceration may have deleterious effects on health, which may be compounded if the necessary community healthcare infrastructure is absent.

### Community behavioral health service capacity

The capacity of local communities to provide behavioral health services varies considerably. Rural communities tend to have fewer mental healthcare providers [3, 48] and fewer outpatient mental health facilities [13], and rural providers are less likely to accept Medicaid [12]. Communities with larger proportions of low-income populations and higher proportions of Black and/or Hispanic residents have reduced access to behavioral services [12, 13, 18, 48]. Only eight percent of the lowest-income communities have access to office-based mental health/behavioral specialist physicians and psychiatrists, compared to 25.3 percent of higher income communities [11]. Limited community behavioral health services restrict the options for how to respond to individuals with behavioral health disorders who commit crimes—even minor offenses like disorderly conduct [15].

The lack of community mental health services, and/or substance use services, has been attributed to deinstitutionalization of mental health services in the 1980s [15] and changes in the sociostructural factors that contribute to inadequate concomitant investment in community-based services. Limited community mental health services restrict the options communities have to respond to individuals with behavioral health disorders who commit crimes [15]. Individuals with mental health and/or substance use disorders may participate in criminal behavior as a result of poverty [15, 29], low self-regulation and control [29], and other offending behavior that is typically unrelated to their mental illness [44]. Individuals with mental health disorders may be arrested due to disorderly conduct or disruptive behaviors. Numerous studies have identified that as hospital [47] and psychiatric bed usage declines [16, 33], incarceration rates tend to increase.

The relationship between community behavioral health capacity and jail utilization is understudied. As explained above, how jail incarceration is associated with adverse behavioral and physical health outcomes has been addressed [24, 34, 65]. However, less is known about the reverse: how behavioral health and associated healthcare access in communities are related to the size of jail populations. A better understanding of the relationship between the amount of local community health and behavioral health services available and jail utilization is needed to further inform program and policy development. The goal of the current study is to bridge the gap in the literature to explore factors related to behavioral health services in the community, as well as the demographics and socio-economic factors, that may predict jail utilization. Drawing on data from the entire population of U.S. counties, this study examines whether the supply of community-based behavioral health services, and public health and socio-economic factors, can predict the size of the jail population. We hypothesize that behavioral health accessibility factors, as well as some demographic and socioeconomic factors of the counties are related to, and can predict, incarceration rate.

## Methods

After variable selection steps, a beta regression model was performed to identify macro-county characteristics that predict of jail population size for all 3,141 U.S. counties. In this study, we examined how demographic, socioeconomic, physical and mental health, and criminal legal system variables relate to jail population per capita in counties across the U.S.

### Data source and measures

Our analysis relied on the most recent data from several sources about the 3,141 counties in the United States to characterize county population and social indicators. Data were collected mainly in 2018 and 2019 but range from 2014 to 2019 (see Table 1 for details). First, the Vera Institute’s incarceration trends database [60] was used to obtain county-level incarceration statistics on the raw count of jail populations. Second, health, economic, social, and demographic information were obtained from the Robert Wood Johnson Foundation (RWJ)’s County Health Rankings & Roadmaps (CHRR), which is compiled from a variety of data sources by the University of Wisconsin [54]. Police data were obtained from the Uniform Crime Report [56, 57]. The most recent collected data on the number of police officers is from 2018. The numbers do not differ substantively per city and county from the reported 2011 data; 2018 version of this variable was not used because it contained a lot of missing data. Therefore, for this variable, the 2011 data, which had fewer missing data, was used. An indicator of whether or not a medical school was present in each county was obtained from the Association of American Medical Colleges [2]. Per capita rates in each county (such as psychiatrists per capita, licensed psychologists per capita, community MH centers per capita, police per capita, and jail population per capita) were calculated using the population counts from the U.S. Census; the latest version of this data was collected in 2018 [55]. Using data from different years was due to the lack of availability of data due to the timing of the extant data (i.e., census data are either actual numbers from 2010 or estimates based on 2010 counts),and we utilized the data compiled in the CHRR since it was identified as the most complete data at the county level. The above data sources were linked using county and state identifiers.

**Table 1:** Description of public health and justice factors used in the models

Variable	Source
**Demographics of the County**	
Size. Indicator variables were created for the three county populations: < 250,000, between 250,000 and 750,000, and over 750,000	U.S. Census Population Estimate﻿s [54]
Percent of population living in a rural part of the county	U.S. Census Population Estimates [54]
Median household income	Small Area Income and Poverty Estimates [54]
Income inequality which reflects the difference between the 80^th^ and 20^th^ income percentiles	American Community Survey, 5-year estimates [54]
High school graduation rate	EDFacts [54]
Percent of population that are African American	U.S. Census Population Estimates [54]
Percent of population that are Hispanic	U.S. Census Population Estimates [54]
**Health Care Related Variables**	
Number of physically unhealthy days or days an individual indicates they were not feeling well	Behavioral Risk Factor Surveillance System [54]
Primary care physician rate based on number of physicians in a county	Area Health Resource File/American Medical Association [54]
Total amount of costs from health care	Dartmouth Atlas of Health Care [54]
Percent of drug treatment services paid by Medicaid	IMS Institute for Healthcare Informatics [1]
Indicator of a medical school in the county	Association of American Medical Colleges [2]
Psychiatrists per capita	American Health Resources File [54]
Licensed psychologists per capita, indicating the total number of licensed psychologists divided by the total county population	American Health Resources File [54]
Community MH centers per capita to indicate outpatient services	American Health Resources File [54]
**Crime-Related Variables**	
Violent crime rate comprised of murder and non-negligent manslaughter, rape, robbery, and aggravated assault per adult population	Uniform Crime Reporting [54]
Police per capita indicating number of police officers divided by the total county population	Uniform Crime Report [56, 57]
**Outcome Variable**	
Jail population per capita, indicating the average daily number of individuals in a jail divided by the total county population	Bureau of Justice Statistics (2015) [60]

### Predictor variable selection steps/procedures

The process to select variables started with a diverse panel of criminologists, psychologists, mental health professionals, biostatisticians, and health economists reviewing available extant data and selecting an original set of potentially important variables based on prior research. Next, machine learning dimension reduction and variable selection methods were applied to select a subset of variables that were indeed important to be included in the main analysis.

In more detail, based on a preliminary review of the county-level characteristics, correlation analysis, and the dimension reduction random forest method [6], 17 variables were identified as the most important variables in predicting county per capita jail population and explained the highest amount of variation of this response variable from the pool of over 34 variables (see [39], and [23], for additional details). These variables are summarized in Table 1. Machine learning least absolute shrinkage and selection operator (LASSO) variable selection technique [26] was then performed, which allowed us to select the 12 most relevant variables among these predictors.

Random forests and LASSO are dimension reduction and variable selection approaches appropriate to use when working with numerous predictor variables. Using a large number of available predictor variables in a regression model is simply not feasible; therefore, for the sake of parsimony of the models and to avoid noisy and overfitted models [37], advanced dimension reduction and variable selection techniques were used prior to fitting the beta regression models. This step helped to prevent multicollinearity in advance of fitting regression models. Multicollinearity occurs with high intercorrelations among two or more predictors, while overfitting occurs when a model is too complex and begins to describe the random error in the data rather than the relationships between variables [37]. Using dimension reduction and variable selection techniques helps select the most important and relevant variables that do not overlap while predicting the response variable. Considering the non-linear nature of the beta regression model, LASSO is one the best methods to select the most relevant variables—which describe the highest percentage of the response variation—for inclusion [49, 67]. Taking this step ensured that predictor variables did not overlap and that the variables were not masked or misrepresented while predicting the outcome variables in the primary analysis.

### Final variables for inclusion in primary analysis

The variable selection procedure resulted in 12 independent/predictor variables to test as predictors of per capita jail population. Predictor variables were converted into percentages, rates, or an index to have comparable scales.

Selected criminal legal variables included: *Police Presence.* The police officer rate per capita was used to indicate a propensity toward crime control in the counites. County- and city- level sworn-in police officers were added together and divided by the total county population. This measures the concentration of police per capita, which may be related to the number of arrests that occur and ultimately the jail population rate. *Crime rates*. Violent crime rates (personal crimes such as murder, assault, rape, and robbery on a per 100,000 rate) were used in the study. We considered using the National Crime Victimization Survey aggregated at the county level to include a county-level indicator of criminal victimization but the data at the county-level was not publicly available or easily retrievable [64]. Crime data used for this study is a suitable proxy for measuring county-level of criminal activity,therefore, the violent crime rate was used within the regression model to indicate the type of crime in a community. The study initially considered including *homicide rate* and *firearm fatality rate*, but these variables had a higher percentage of missing information for smaller counties and missing data rates of 76 and 28 percent respectively for all counties; additionally, these variables were statistically correlated with violent crime rate. Therefore, as expected, such variables were not selected by the variable selection method to be used within the beta regression model.

Demographic variables entered into the second round of variable selection included county rurality or percent of population living in a rural area, the population size of each county, high school graduation rate, and percent of minority population, which included percent of population African American and percent of Hispanic population. County size was used as an indicator variable with three categories representing population size of each county. The U.S. counties were categorized in three population sizes: Small counties have a population less than 250,000 (*N* = 2,880), medium-sized counties have with a population between 250,000 and 750,000 (*N* = 186), and large counties have populations greater than 750,000 (*N* = 75). Data on small counties tend to contain more missing demographic and crime-related data elements. The size of the county is featured in all parts of the analyses by including this variable in the model, so it is controlled for while exploring the other variables that predict jail population per capita. Race was found to be collinear with most of other predictors, and therefore excluded without negatively affecting the fit of the model [37]. To ensure that the model would not be negatively affected by excluding the race variable, the relationship between the race factor and other variables of our model was studied. The racial characteristics alone did not explain a high amount of the response variation, nor did they improve the model fit. The addition of race to the model in combination with other variables not only masked the true relationship between predictors and the response, it also made the model estimates unstable as a result of strong mutual collinearity. However, the variance in race was explained through other variables included in the model due to the high collinearity. The variables kept in the model were substantially more predictive of the response compared to the racial variables and led to an improved model fit.

Median household income and income inequality were originally considered as candidates for measuring the socio-economic status of the county. However, correlation analysis showed that median household income in the county was correlated with income inequality as well as other county-demographic variables such as education-level. The machine learning variable selection models suggested including only one and preferred income inequality as a more important variable to represent the socio-economic status of the counties in the model, instead of other income-related variables to successfully predict the jail population rate. Removing the median household income and racial characteristics of the counties did not result in the loss of any information, but improved the model fit statistics.

Variables to account for health care-related issues and resources in the counties were selected for the regression model based on their relationship to the response (i.e. jail population size) using machine learning techniques including number of physically unhealthy days, primary care physician rate, total amount of costs from health care, percent of drug treatment services paid by Medicaid, and the presence of a medical school within a county. Variables representing behavioral health capacity and accessibility in a county selected by the variable selection techniques were psychiatrists per capita, licensed psychologists per capita, and community MH centers per capita, which includes psychiatrists, psychologists, counselors, nurse practitioners and social workers. These variables were not highly correlated and did not result in any multicollinearity issues; therefore, they all were kept as predictors in our model.

The dependent variable in the primary analysis was jail population per capita. The jail population per capita consists of the average number of individuals in jail on any given day as a percentage of the population of the county.

### Statistical analysis

Statistical analyses were performed in SPSS 25﻿ [21] and R [40]. A significance level of $\alpha =.05$ was used and all hypothesis tests were 2-sided. After the intial steps of checking the correlations and summary statistics among jail population per capita and the independent variables and selecting the important predictors, a beta regression model was fitted to identify macro-county characteristics that predict of jail population size among all 3,141 U.S. counties. To draw generalizable conclusions about the factors that are related to the county-level jail population per capita across the U.S., the entire population of U.S. counties was studied. Beta regression is the most appropriate predictive model here since the dependent variable was a ratio variable, with values restricted to 0 and 1, that follow a beta distribution, not a normal distribution [14]. The 0 to 1 interval restriction on the dependent variable and the non-normal nature of the response variable made linear regression models inappropriate,instead, the use of the maximum likelihood estimation technique within beta regression models were naturally adept for accurately modeling rates. Beta regression models the association of predictors to changes in mean and variance simultaneously, and it performs well on transformed percentage/ratio data [36].

## Results

Summary statistics, correlation analysis, dimension reduction and relevant variable selection methods, and then statistical models demonstrated the importance and relevance of the size of the county and how it accounts for a statistically significant difference in jail utilization rates, as shown in Table 2. Small counties have different predictors of the jail population per capita than the medium- and large-sized counties. Small counties tend to be more likely to be located in rural communities, have a lower median household income, are not racially diverse, have a higher number of unhealthy days and do not have easier access to health care. This is different than larger-sized counties and, although these counties have lower crime rate and police per capita than larger counties, they have higher jail population per capita.

**Table 2:** Description of key variables by county size

Variable	Large county (*n* = 75, 2.4%)		Medium county (*n* = 186, 5.9%)		Small county (*n* = 2,882, 91.7%)	
	Mean	Range	Mean	Range	Mean	Range
**Demographics of the county**						
Percent of population living in a rural area	2.83	0 to 18.39	10.98	.18 to 31.03	48.08	0 to 100
Median household income	65,439	41,514 to 115,518	63,459	39,629 to 134,609	51,817	30,467 to 10,8635
Income inequality	4.92	3.76 to 7.19﻿	4.51	3.46 to 5.71	4.43	3.13 to 6.88
High school graduation rate	81.38	66.28 to 93.59	82.81	59.96 to 93.2	85.54	32.56 to 97.5
Percent of population that are African American	16.29	1.60 to 62.49	11.49	.92 to 54.06	7.04	.15 to 80.74
Percent of population that are Hispanic	23.52	2.03 to 82.2	14.53	1.13 to 58.88	9.03	.70 to 83.75
**Health care related issues**						
Number of physically unhealthy days	3.55	2.44 to 4.57﻿	3.59	2.32 to 4.63	3.72	2.40 to 5.76
Primary care physician rate	87.05	42.02 to 158.53	84.52	17.46 to 177.50	61.68	0 to 228.48
Total amount of costs from health care	9,710	7,902 to 13,762	9,253	7,294 to 11,817	8,995	6,284 to 13,078
Percent of drug treatment services paid by Medicaid	19.89	4.4 to 49.5	20.41	5.3 to 49.5	22.49	4 to 49.5
Percent of counties with a medical school	70.7%		22.04%		.9%	
Psychiatrists per capita	.0002289	.000028 to .001313	.0002289	.0000167 to .0007414	.000039	0 to .001423
Licensed psychologists per capita	.0005042	.00006111 to .002134	.0004043	.00003093 to .002011	.00014	0 to .01136
Community MH centers per capita	.000000612	0 to .0000068	.000000523	0 to .00000867	.0000004	0 to .000178
**Crime issues**						
Violent crime rate	8,223.61	1,010 to 42,555	1,711.33	277.33 to 5,525	202.99	.33 to 1,710
Police per capita	2.0	.61 to 4.34	1.67	.73 to 3.55	1.5	.03 to 4.63
**Outcome variables**						
Jail population per capita	.0028	.0005 to .0074	.0030	.00001 to .008	.0033	0 to .0165

Table 3 shows the results of the beta regression for predicting per capita jail population. The following county health-related factors significantly predicted a higher jail population per capita: more average physically unhealthy days in the past month (30 days) (z-score = 8.6), fewer psychiatrists per capita (-2.16), higher health care costs (5.71), and a lower percent of drug treatment paid by Medicaid (-3.66). Among demographic factors, a lower high school graduation rate (-4.05) and small county size compared to medium and large counties (-2.66 and -2.71 respectively) predicted a higher per capita jail population. The only significant crime-related factor was police per capita (8.74). More police per capita predicted a higher per capita jail population. Income inequality, primary care physician rate, community MH centers per capita, and violent crime rate did not significantly predict jail population per capita.

**Table 3:** Beta regression model for predicting jail population per capita

	Estimate	standard error	Z-score	*p*-value
(Intercept)	-5.263	0.016	-332.413	< .001
Income inequality	0.009	0.015	0.554	0.580
High school graduation rate	-0.054	0.013	-4.046	< .001**
County size Medium vs Small	-0.156	0.059	-2.659	0.008**
County size Large vs Small	-0.335	0.123	-2.712	0.007**
Poor physically unhealthy days	0.144	0.017	8.616	< .001**
Primary care physician rate	0.007	0.018	0.405	0.686
Health care costs	0.089	0.016	5.701	< .001**
Percent of drug treatment paid by Medicaid	-0.049	0.013	-3.664	< .001**
Psychiatrists per capita	-0.039	0.018	-2.159	0.031*
Community MH centers per capita	0.009	0.014	0.604	0.546
Violent crime rate	-0.011	0.019	-0.59	0.555
Police per capita	0.150	0.017	8.736	< .001**

** represents significance at the 0.01 level in a two-tailed test; * represents significance at the 0.05 level in a two-tailed test

Note 1: Considering that beta regression models use a log transformation when modeling the response variable, estimates/coefficients need to be exponentiated before reporting/interpreting the strength to which they contribute to explaining the response variation

Note 2: The z-scores indicate the strength of the relationship between each predictor and the outcome variable while holding everything else constant, and the p-value column is used to evaluate whether each variable plays a statistically significant role in predicting the outcome. The direction of the relationship is designated by whether the z-score is negative or positive

To be more specific, for psychiatrists per capita, the odds ratio (OR) is obtained from the beta regression coefficient in Table 3, which is exp(-0.039) or ${e}^{-0.039}$, or commonly referred to as OR = 0.96. The odds ratio indicates that each one-unit increase in psychiatrists per capita is predicted to reduce the ratio of jailed to non-jailed population per capita by 4%. Similarly, for percent of drug treatment paid by Medicaid, OR = exp(-0.049) or 0.95, which indicates that each one percent increase in the percent of drug treatment paid by Medicaid is expected to reduce the ratio of jailed to non-jailed population per capita by 5%. The relationship is the opposite for the police per capita; it has an odds ratio of exp(0.150) = 1.16, which indicates that a one unit increase in the police per capita, is expected to have a relative increase of 16% in ratio of jailed to non-jailed population per capita. Odds ratios for the other predictors are obtained in the same way and have the same interpretation.

Violent crime rate, income inequality, primary care physician rate, and number of community mental health centers per capita were not significant predictors of per capita jail population when the effect of community treatment service capacity and county demographic factors were accounted for.

## Discussion

Using statistical and machine learning methods, this study explored macro-level health, legal system, and demographic factors to identify predictors of the size of the jail population in U.S. counties. Regardless of the size of the county, the models revealed insights into contributing factors that are seldom identified in studies of jail populations. In this study, one county-level health factor emerged as important factor influencing per capita jail population: more physically unhealthy days within the past 30 days predicted a higher per capita jail population. Other relevant factors related to a higher jail population per capita are lower behavioral health capacity (measured by per capita rate of psychiatrists) and less access to services (higher health care costs and lower percent of drug treatment paid by Medicaid). It is notable that these behavioral healthcare access and affordability factors were better predictors of per capita jail population than violent crime rate (while controlling for county size). The fear of violent crime and the criminalization of many behaviors are typical crime control responses that are more punitive. That is, in the era of mass incarceration, as the violent crime rate in a community increases, we would expect that to be a driver of incarceration in local communities due to our “get tough” policies [17]. Therefore, while it might be expected that legal system-related factors would be relevant, the beta regression results suggest that in the presence of health-related and demographic variables, the violent crime rate is not a significant predictor of jail population. In fact, only one justice variable (a greater concentration of police) predicted larger jail populations. As it can be seen on Table 3, for every additional police officer (per capita) in a county, the ratio of jailed population to non-jailed population per capita is expected to increase by roughly 16 percent.

Small counties had a much higher rural population, lower median household income, less racial diversity, higher number of unhealthy days, and less health care accessibility/availability, compared to the medium- and large-sized counties. Counties with smaller populations also had higher jail population per capita despite lower crime rate and fewer police per capita. Medium-sized counties had a roughly 15 percent lower jail population per capita, and large counties a 40 percent lower jail population per capita than did small counties. Although other factors may be at play, lower mental health service accessibility in smaller counties may be an important driver of their higher incarceration rates. Our prior research has described how jails become mental health or substance use care of last resort when police do not have other places to take someone [22].

This paper examined an understudied area of mass incarceration—the degree to which community behavioral health service capacity predict the size of the jail population. The tendency of policies and programs is to treat criminal legal and behavioral health care as separate systems, each with different and independent investment decisions. This creates a schism in policymaking on how to best address health and behavior needs of counties’ populations. Attention to how health care policies and practices are related to the size of the jail can bridge an important policy and program gap. Greater concentrations of police and lower rate of mental health providers are both associated with greater jail use, and this argues for considering these disparate systems together in budget, policy, and programs processes. Services that are within geographical reach of individuals (i.e., within two miles from an individual’s residence) are more likely to reduce recidivism [19, 20]. Further investments in behavioral health care could reduce the use of incarceration for individuals with behavioral health disorders, as well as improve health equity.

Greater investment and funding of community health and behavioral health services offers the justice system more options for preventing or responding to a range of disruptive and criminal behaviors that may result in jail detention. Programs that divert individuals from jail to treatment are not feasible where treatment services are lacking. The current trend to offer diversion programs for individuals with mental illness has met with resistance when police have few options other than jail [4, 9]. There is a need to attend to expanding behavioral health services to reduce overuse of the legal system including jails.

### Limitations

This study relied upon the most recent extant census data, surveys, and databases on justice and health factors. One limitation is that the extant data came from various years given availability of the data, but most were published within a five-year range except for police per capita. As explained above, we checked the most recent data of police officer rate per capita from 2018 and it was very similar to the 2011 file, except the 2011 data had fewer missing data. The analysis also was constrained by not including potential access to services in adjacent counties, however, this may be difficult to do because many agencies have residency restrictions on access to substance use and/or mental health services for county and/or state funded services.

The statistical and machine learning tools selected the predictors that were available in these data sets. Although there may be other factors that could potentially predict the county-level incarceration rate in the U.S., the study was constrained to the extant data. Random forests and LASSO dimension reduction and variable selection techniques were used to identify predictor variables. This means that other social determinants of health such as food availability, employment factors, and physical environment were considered but not selected within the variable selection step. However, there are important variables that we could not use because they are not available in extant data sets including county services such as legal representation, social supports, homelessness, and diversion programs for individuals with mental illness. We were also unable to include a county-level indicator of criminal victimization because county-level National Crime Victimization Survey data was not publicly available or easily retrievable.

Finally, considering that higher rates of incarceration occur in communities with larger proportions of racial and ethnic minority group members and/or heightened economic deprivation [28], and (when holding other variables constant) income differences between Whites and Blacks and minority population sizes is related to jail sizes in large cities [8], race was initially selected as a potentially important factors by our expert team. However, it was excluded within the variable selection procedure due to its collinearity with most of the other predictors. This means that since race is related to many other factors in health care, legal system, and community socioeconomic features, adding race masked various effects that were strongly related the jail population per capita,therefore, we did not include race in the models. Model fit statistics, as well as the fact that the variance of race was already explained in the model by collinear variables, provide the appropriate justification for the statistical methods used to handle this issue (see [37]. This study therefore examines other factors that contribute to the jail population.

## Conclusions

This study demonstrates that communities with the greater amount of both mental health services, and affordable services, have significantly lower incarceration rate per county. This may indicate that counties with greater accessibility and affordability of mental health care services are able to respond to a range of mental and behavioral health issues without relying on incarceration. Counties with a better accessibility and affordability of health care and behavioral health services, including the affordable drug treatment services (as a result of Medicaid funding), may be able to respond more appropriately to the health needs of the population while reserving incarceration for those engaging in more serious criminal behavior. Healthcare policy efforts may help address the overuse of county-level incarceration. Based on our results, smaller counties that are health resource-challenged, and those that are more policed, have larger per capita jail populations. Attention to improving mental health, substance use, and healthcare access generally may have a collateral impact on increasing the opportunity to use diversion and/or alternatives to incarceration to decrease the demand for jail beds.

Considering the higher rate of incarceration in smaller counties and the fact that counties with a smaller population size have fewer resources, allocating those resources to physical and behavioral health centers may help to reduce jail populations. This may be the best use of scarce resources given that mental health and substance use care have been found to be more cost-effective at promoting recovery and reducing subsequent crime than incarceration [58, 59]. The collective goals of improving public health and decarceration efforts could be met by addressing behavioral health resource gaps and providing more accessible and affordable health and mental health services to county residents. More research is necessary to determine specific treatment and support services that help reduce incarceration rate, and how more of these services can be introduced to US communities that have a deficit of these services.

## Data Availability

The datasets used and/or analyzed during the current study are available from the corresponding author on reasonable request.

## Abbreviations

CHRR: County health rankings & roadmaps
LASSO: Least absolute shrinkage and selection operator
MH: Mental health
RWJ: Robert wood johnson foundation
